# Aberrant immunoexpression of p53 tumour-suppressor and Bcl-2 family proteins (Bcl-2 and Bax) in ameloblastomas and odontogenic keratocysts

**DOI:** 10.4317/jced.59769

**Published:** 2023-02-01

**Authors:** Enrico Escobar, Fernán Gómez-Valenzuela, Cristian Peñafiel, Eduardo Chimenos-Küstner, Ricardo Pérez-Tomás

**Affiliations:** 1 Department of Oral Pathology and Medicine, Faculty of Dentistry, University of Chile, Santiago, Chile; 2 Faculty of Medicine, Pontificia Universidad Católica de Chile, Santiago, Chile; 3 Department of Odonto-Stomatology, Faculty of Medicine and Health Sciences, University of Barcelona, Barcelona, Spain; 4 Department of Pathology and Experimental Therapy -Bellvitge, Faculty of Medicine and Health Sciences, University of Barcelona, Barcelona, Spain

## Abstract

**Background:**

The growth of ameloblastomas (odontogenic tumours) and odontogenic keratocyst (OKC) (developmental cyst) is associated with the expression of proteins related to cell survival and apoptosis. Bcl-2-associated protein X (Bax) and the tumour suppressor protein p53 collectively promote p53-mediated apoptosis. This study aimed to assess the immunohistochemical expression of p53, Bcl-2 and Bax in conventional ameloblastoma (CA), unicystic ameloblastoma (UA) types, and OKC sporadic (OKC-NS/S) and syndromic (OKC-NBSCC).

**Material and Methods:**

Paraffinized blocks of CA (n=18), UA (n=15), OKC-NS/S (n=18) and OKC-NBSCC (n=15) fixed in 10% formalin were used. After diagnosis, tissue specimens were stained by immunohistochemistry for p53, Bcl-2 and Bax marker. Stained cells were randomly counted in five high power fields. The data analysis was performed via Shapiro-Wilk test, ANOVA with Tukey’s multiple comparisons or Kruskal-Wallis with Dunn’s multiple comparisons. Statistical significance was defined as *p*<0.05.

**Results:**

We did not observe differences between p53 expression in CA, mural UA (MUA), intraluminal/luminal UA (I/LUA), OKC-NS/S, and OKC-NBSCC (19.69%, 18.74%, 16.76%, 12.35% and 9.04%, respectively). Similar results were recognized for Bax expression in CA, MUA, I/LUA, OKC-NS/S, and OKC-NBSCC (33.72%, 34.95%, 22.94, 21.58% and 20.76%, respectively). However, we recognized significant differences between Bcl-2 expression in OKC-NS/S vs MUA, OKC-NS/S vs I/LUA, OKC-NS/S vs CA, OKC-NBSCC vs MUA, OKC-NBSCC vs I/LUA, and I/LUA vs CA. P53, Bcl-2 and Bax levels were higher in mural morphological areas versus intraluminal and luminal morphological areas in UA.

**Conclusions:**

There is a tendency for an increased expression of p53, Bcl-2, and Bax proteins in CA, and mural proliferation of UA, compared to lesions with a cystic morphology, which could be associated with a local aggressive behaviour.

** Key words:**p53, Bcl-2, Bax protein, apoptosis, odontogenic tumour, odontogenic cyst.

## Introduction

Apoptosis represents a form of programmed cell death that plays a role in the homeostasis of tissues (1). However, alterations in various cell signalling pathways can lead to dysregulation of apoptosis and promote tumourigenesis (1).

There are two main apoptotic pathways: the extrinsic and the intrinsic or mitochondrial pathway (1). P53, Bcl-2 and Bax proteins are among the most studied effectors during the intrinsic pathway of apoptosis. P53 is a primary orchestrator of the cellular response to a broad array of stress types by regulating apoptosis, cell cycle arrest, senescence, DNA repair and genetic stability (2). P53 participates directly in the intrinsic apoptosis pathway by interacting with the multidomain members of the Bcl-2 family. The Bcl-2 family determines the commitment of cells to apoptosis (2,3). The Bcl-2 protein is an anti-apoptotic molecule that modulates the mitochondrial release of cytochrome c, and the interaction of apoptosis activating factors with caspase 9 and Bax. P53-dependent signals, including the induction of Bax and direct inhibition of Bcl-2, synergize with p53-independent signals to antagonize Bcl-2 function and promote apoptosis (3). Bax, a pro-apoptotic member of Bcl-2 family, has been considered as a potential tumour suppressor (2,3). P53 regulates the transcription of Bax gene and the loss of p53 function causes diminished expression of Bax protein (4).

Conventional ameloblastoma (CA), unicystic ameloblastoma (UA) and odontogenic keratocyst (OKC) are common lesions in the jaws. CA is the most frequent of the benign epithelial odontogenic tumours(5). UA represents a distinct type of ameloblastoma regarded as a neoplastic cyst (5). Likewise, UA is typified according to cellular growth as luminal UA (LUA), intraluminal UA (IUA), and mural UA (MUA). In LUA and IUA, the cystic wall is not invaded by growth of the luminal lining (5). In contrast, MUA presents a compromise of the cystic wall with involvement towards a locally aggressive behaviour (5).

The current 2022 World Health Organization (WHO) odontogenic tumours classification identifies OKC as a nonneoplastic cyst with the potential for aggressive behaviour and a tendency for local recurrence (6). Moreover, OKC development is related to naevoid basal cell carcinoma syndrome (NBCCS) (6). The biologic behaviour of syndromic OKC (OKC-NBCCS) has been described as more aggressive and with higher recurrence rates compared with OKC non-syndromic or sporadic OKC (OKC-NS/S) (7).

The expression of p53, Bcl-2 and Bax proteins has been studied in various pathological lesions, especially malignant neoplasms (8). Nevertheless, research about the expression of p53, Bcl-2 and Bax proteins in odontogenic lesions is scarce, particularly for samples from Latin-America. Interestingly, the expression of proteins related to tumour-suppression genes (p53), and Bcl-2 family could be independent of the morphological characteristics of solid and cystic odontogenic lesions.

In this fashion, this study aimed to analyse and compare the expression of p53, Bcl-2 and Bax proteins in CA, UA, OKC-NS/S, and OKC-NBCCS as odontogenic lesions with a similar clinical-biological behaviour, but morphologically different, by immunohistochemical study.

## Material and Methods

Sample selection. A sample of 66 cases was collected. All cases and demographic data were retrieved from the Pathological Anatomy Service, University of Chile, from 1997 to 2016. Haematoxylin-eosin-stained slides were studied by two pathologists and re-evaluated and diagnosed according to 5th edition of the WHO (2022). Previously, a Cohen’s Kappa (κ) coefficient was performed by two calibrated observers (κ-value= 0.81±0.12 with a 95% confidence interval of 0.56 to 1.00) for the histological diagnosis of CA, UA and OKC. The cases were distributed into four categories: CA (n=18), UA (n=15) according to histologic types: luminal and/or intraluminal UA (I/LUA) (n=7), and luminal and mural and/or intraluminal UA (MUA) (n=8), and OKC (n=33) according to OKC-NS/S (n=18) and OKC-NBCCS (n=15). Two subcategories for UA were considered according to their dissimilar clinicopathological behaviour and treatment, especially for UA with mural proliferation. The diagnosis for NBCCS was made according to Bree and Shah’s criteria (9). Lesions with an inflammatory infiltrate (moderate to intense) were excluded following the criteria of Clark *et al*. (10). Likewise, recurrent lesions or lesions obtained after decompression therapy were excluded.

Immunostaining method. Four-μm thick sections from paraffin-embedded blocks in a Reichert-Jung® Biocut Microtom (Mod.1130/Biocut) were placed on positively charged slides (CellPath Ltd.). Cases were deparaffinized in xylene for 20 minutes (min) and hydrated in graded ethanol. Next, the slides were placed on distilled water for 10 min. Deparaffinized slides were placed in PBS (pH=7.4±0.2). Subsequently, the slides were immersed in 10mM citrate buffer solution (pH=6.0) for antigen retrieval and were transferred to Oster® Food Steamer until the boiling point. The cases were maintained for 20 min and left for cooling until 21°C. Internal peroxidase activity was blocked using 3% hydrogen peroxide and Mouse/Rabbit Immunodetector Peroxidase Blocker Solution (BSB0003, Bio SB) for 20 min each at room temperature. Primary monoclonal antibodies (Table 1) from Bio SB Inc. were incubated overnight at 4 °C for mouse anti-human P53 (BSB5842, clone: DO7, Ready-To-Use); mouse anti-human Bcl-2 (BSB5075, clone: BCL2/A4, Concentrated), and rabbit anti-human Bax (BSB6079, clone: E63, Ready-To-Use).

**Table 1:**
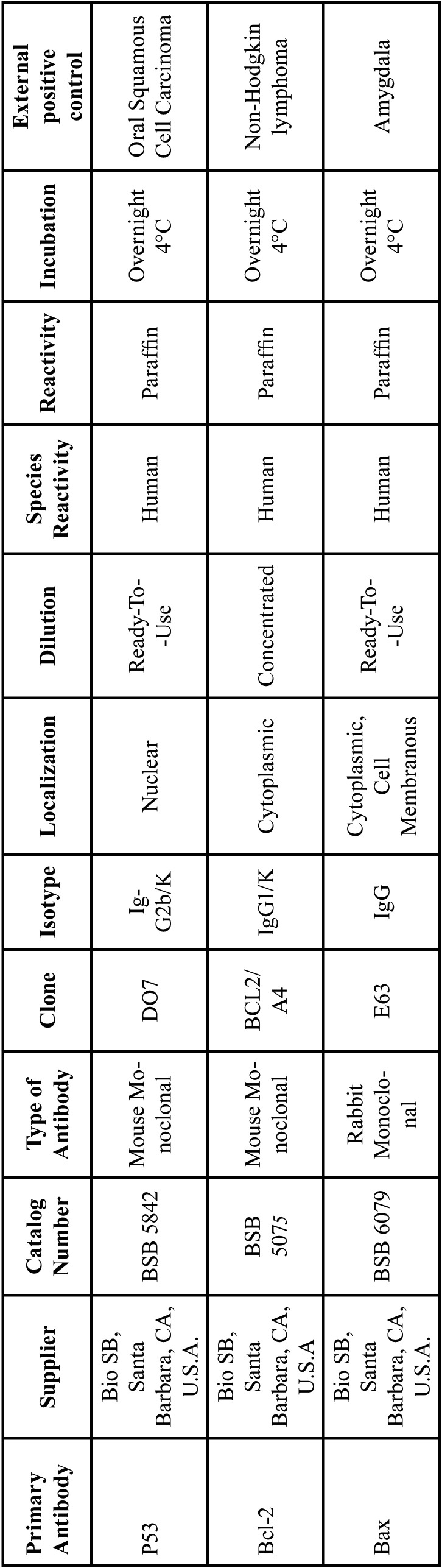
Details of primary antibodies employed.

**Figure 1:**
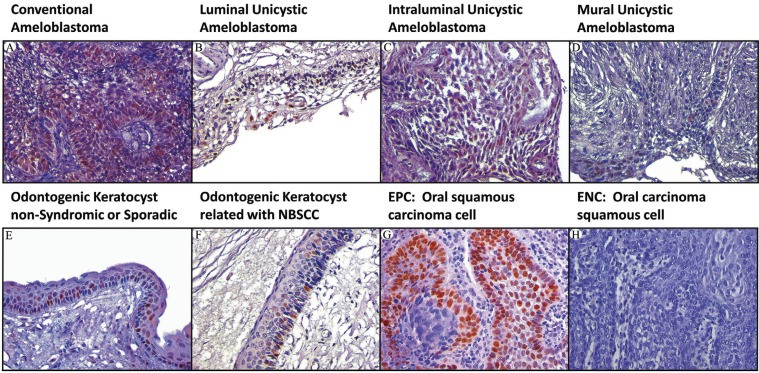
Immunohistochemical protein expression of p53 (400x). (A) conventonal ameloblastoma; (B) luminal unicystic UA; (C) intraluminal UA; (D) mural UA; (E), OKC non-syndromic or sporadic; (F), OKC related NBSCC; (G), EPC: oral squamous cell carcinoma; (H), ENC: oral squamous cell carcinoma. Abbreviations: UA: unicystic ameloblastoma; EPC: external positive control; ENC: external negative control; OKC: odontogenic keratocyst; NBSCC: naevoid basal cell carcinoma syndrome.

**Figure 2:**
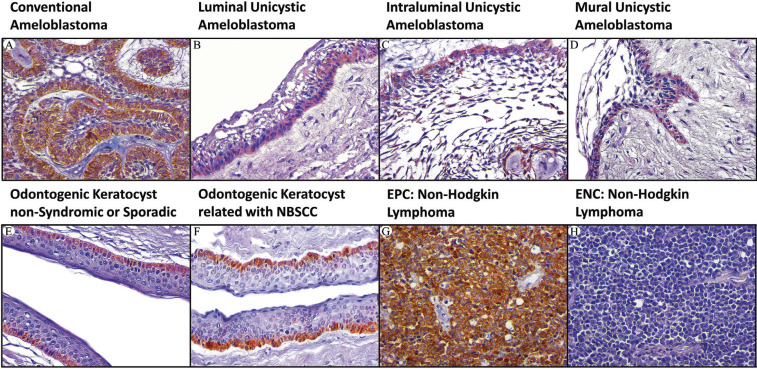
Immunohistochemical protein expression of Bcl-2 (400x). (A) conventional ameloblastoma; (B) luminal unicystic UA; (C) intraluminal UA; (D) mural UA; (E), OKC non-syndromic or sporadic; (F), OKC related NBSCC; (G), EPC: non-Hodgkin lymphoma; (H), ENC: non-Hodgkin lymphoma. Abbreviations: UA: unicystic ameloblastoma; EPC: external positive control; ENC: external negative control; OKC: odontogenic keratocyst; NBSCC: naevoid basal cell carcinoma syndrome.

**Figure 3:**
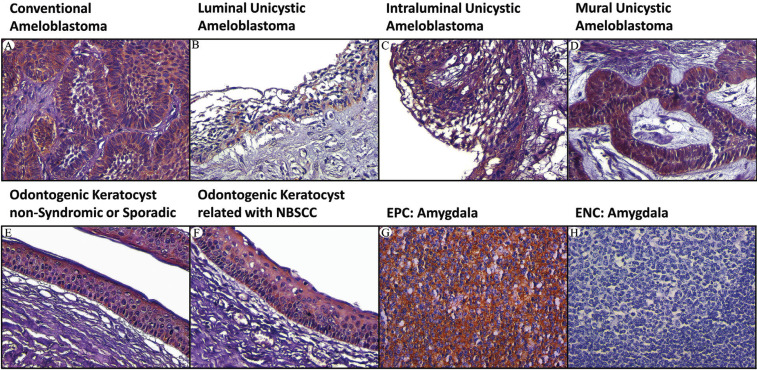
Immunohistochemical protein expression of Bax (400x). (A) conventional ameloblastoma; (B) luminal unicystic UA; (C) intraluminal UA; (D) mural UA; (E), OKC non-syndromic or sporadic; (F), OKC related NBSCC; (G), EPC: amygdala; (H), ENC: amygdala. Abbreviations: UA: unicystic ameloblastoma; EPC: external positive control; ENC: external negative control; OKC: odontogenic keratocyst; NBSCC: naevoid basal cell carcinoma syndrome.

Next, the cases were incubated with Mouse Immuno-Detector Biotin-Link and mouse/rabbit Immuno-Detector HRP Label (BSB0003) for 20 min each at room temperature. PBS was used as a wash buffer for 30 min when required between each stage. Peroxidase reaction was performed with chromogen 3,3′-diaminobenzidine reagent. The cases were slightly counterstained with Harris’s haematoxylin solution for one minute. Oral squamous cell carcinoma (Fig. 1G), non-Hodgkin lymphoma (Fig. 2G) and amygdala (Fig. 3G) were used as a positive external control for p53, Bcl-2 and Bax proteins, respectively. Immunostaining of stromal lymphocytes was utilized as internal positive control for Bcl-2 and Bax proteins. Negative external controls (Figs. 1H,2H) (Fig. 3H) were the same samples of external positive controls incubated with phosphate-buffered saline (PBS; PBS-9990, BION) instead of primary antibodies.

Immunohistochemical scoring. Odontogenic epithelial cell (OEC) immunostaining was quantitatively assessed by estimating the percentage of stained cells per high-power field (HPF) with magnification of 400x. In UA, quantification of p53; Bcl-2 and Bax immunostaining considered to I/LUA: lining epithelium and intraluminal proliferation, and for MUA: exclusively proliferation mural. Moreover, areas characterized morphologically as luminal, intraluminal, and mural were quantified in each case of UA. Five photomicrographs were captured with a digital camera in an optical microscope (Olympus BX41, Tokyo, Japan) for each case in areas with abundant OEC by HPF. Microphotographs were taken and processed with the LEICA application suite software program (v4.7.1). All images were stored in TIF format. Cells were counted by two independent observers using the ImageJ free software. P53 immunoexpression was assessed for the nuclear staining, instead, Bcl-2 and Bax protein immunoexpression was assessed for the cytoplasmic staining. Stained and total number of OEC were counted in five HPF. The number of positive cells for immunoreactivity was counted and calculated as a percentage relative to the total cells. Previously, we performed the Bland–Altman test (a bias of 4.86±4.61 with a 95% confidence interval from -4.17 to 13.88) to analyse the interobserver agreement of the immunoexpression count.

Statistical analysis. Statistical analysis was performed using R Statistical Software (v4.1.2; R Foundation for Statistical Computing, Vienna, Austria). First, we conducted a heteroskedasticity examination. Next, we analysed the data distribution using the Shapiro-Wilk test. Consequently, we performed statistics using one-way ANOVA with Tukey’s multiple comparison test or Kruskal-Wallis with Dunn’s multiple comparisons with Holm’s correction. Last, we conducted Spearman correlation analysis to observe potential correlations. Statistical significance was defined as *p*<0.05.

## Results

Clinical-demographic characteristics. The clinical-demographic features are summarized in Table 2.

**Table 2:**
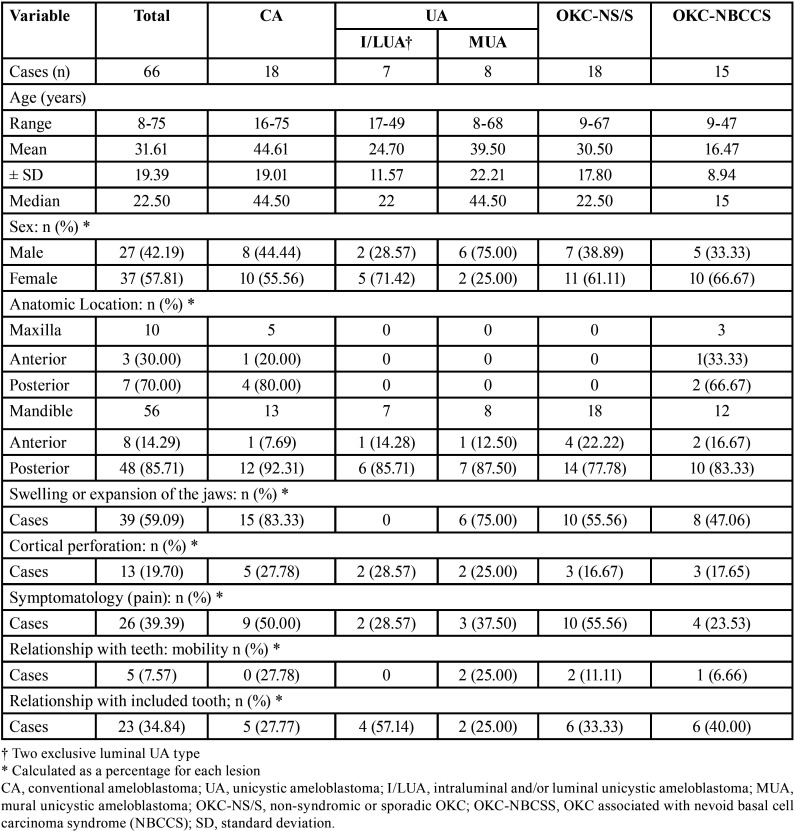
Clinical-demographic characteristics of the involved patients and distribution of the lesions by anatomic location.

Males and females had a similar prevalence. The mean age of prevalence of all studied lesions was 31.61 years with a median of 22.50 years. OKC-NBCSS was the earliest average diagnosis (16.47 years). The location in the mandible (n=56) was significantly greater than those observed in the maxilla (n=10), particularly in posterior areas of CA and UA. Overall, SA was the most symptoms-related lesion.

P53 protein expression. We observed a higher p53 expression in CA, MUA, and I/LUA than OKC-NS/S and OKC-NBSCC; however, no statistical differences were found between these groups (Table 3, A). Likewise, no statistical differences were observed among luminal, intraluminal, and mural areas of UA for p53 expression. In CA, peripheral ameloblast-like cells and stellate reticulum-like cells showed positive brown nuclear p53 immunostaining (Fig. 1A). In UA, positive brown nuclear p53 immunostaining was detected in basal (ameloblast-like) and suprabasal (stellate reticulum-like) layers (Fig. 1B-D). In intraluminal and mural areas of UA, positive nuclear p53 immunostaining was observed both in peripheral ameloblast-like cells and stellate reticulum-like cells (Fig. 1B-D). In OKC-NS/S and OKC-NBCSS, positive brown nuclear p53 immunostaining was detected both in basal and suprabasal compartments of the epithelial lining (Fig. 1E-F).

**Table 3:**
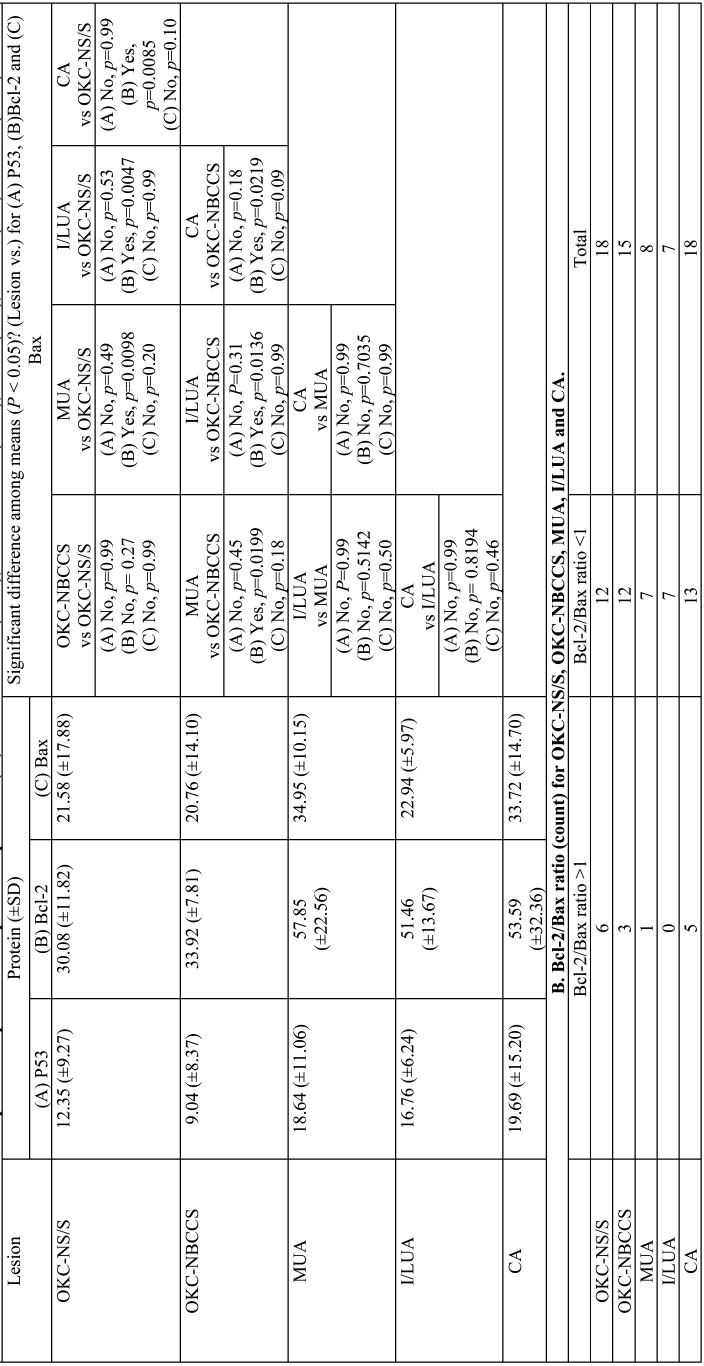
A. Mean ± SD and comparison of p53 and Bax protein expression (%) for OKC-NS/S (n=18), OKC-NBCCS (n=15), MUA (n=8), I/LUA (n=7) and CA (n=18).

Bcl-2 protein expression. Statistical differences were recognized between OKC-NS/S vs MUA, OKC-NS/S vs I/LUA, OKC-NS/S vs CA, OKC-NBSCC vs MUA, OKC-NBSCC vs I/LUA, and I/LUA vs CA. Bcl-2 immunostaining in CA was cytoplasmic and mainly located in peripheral ameloblast-like cells (Fig. 2A). In UA, predominant staining was also observed in ameloblast-like cells (Fig. 2B-D). In OKC-NS/S and OKC-NBCSS, immunostaining was cytoplasmic and localized to the basal cells of the epithelial lining and sometimes to the parabasal cells. The cells of the suprabasal compartments were negative for the immunoexpression of the Bcl-2 protein (Fig. 2E-F).

Bax protein expression. We observed a higher Bax expression in CA and MUA than OKC-NS/S and OKC-NBSCC; however, no statistical differences were found between these groups (Table 3A). Likewise, no statistical difference was found among luminal, intraluminal, and mural areas of UA for Bax expression. Bax immunostaining in CA was cytoplasmic and located in peripheral ameloblast-like cells and stellate reticulum-like cells (Fig. 3A). In UA, positive brown cytoplasmic Bax immunostaining was detected mainly in basal (ameloblast-like) and suprabasal layers (stellate reticulum-like) (Fig. 3B-D). In intraluminal and mural areas of UA, positive cytoplasmic Bax immunostaining was observed both in peripheral ameloblast-like cells and stellate reticulum-like cells (Fig. 3B-D). In OKC-NS/S and OKC-NBCSS, a positive brown cytoplasmic Bax immunostaining was observed both in basal and suprabasal epithelial compartment (Fig. 3E-F).

Protein immunoexpression comparison. The summary of the mean of P53, Bcl-2 and Bax immunoexpression and its comparison between OKC-NS/S, OKC-NBCSS, CA, MUA and I/LUA are presented in Table 3, A and between morphological areas of UA are presented in Table 4, A. Spearman’s test showed a non-statistical correlation between p53 and Bax in the lesions analysed (rho-values for: MUA= 0.37; I/LUA= -0.26; OKC-NS/S= 0.17; OKC-NBCSS= 0.001; and CA=0.25).

**Table 4:**
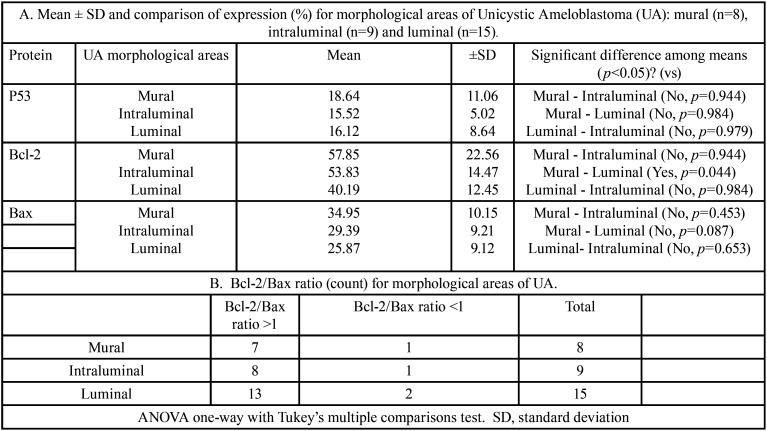
A. Mean ± SD and comparison of expression (%) for morphological areas of Unicystic Ameloblastoma (UA): mural (n=8), intraluminal (n=9) and luminal (n=15).

Comparison of Bcl-2/Bax ratio. We observed that the Bcl-2/Bax ratio classified according to higher Bcl-2 expression (ratio >1) or higher Bax expression (ratio <1) was similar between OKC-NS/S, OKC-NBCSS, CA, MUA, and I/LUA (Table 3B). However, we observed a trend of Bcl-2/Bax ratio >1 in all morphological areas of UA (Table 4B).

## Discussion

The evasion of apoptosis induces tumourigenesis and tumour progression. The p53 gene is frequently mutated in various human tumours (11). P53 interacts with Bcl-2 and neutralizes their inhibitory effects on Bax, as well as p53 stimulates the expression of proapoptotic Bcl-2 family, including Bax (12). Indeed, Bax is a downstream effector of p53-induced apoptosis and is transcriptionally regulated by p53 (11). The positive correlation between the p53 activity (wild type p53) and Bax (4) is based on various p53-responsive elements in the Bax gene promoter (13).

The immunodetection of the p53 protein presents the following considerations: wild-type-p53 protein has a very short half-life in normal cells and is practically undetectable by immunohistochemical staining (13). However, p53 inactivated through mutations, deletions or binding to other proteins results in a P53 protein accumulation that immunohistochemistry can detect (14).

Increased expression of p53 protein has been related to increased proliferative potential, which is involved in tumoural development and progression in OKC, UA and SA (15). In our study, the means of p53 protein immunoexpression were higher in SA and MUA versus OKC NS/S and OKC-NBSCC. However, no statistically significant differences were observed, agreeing with other studies that compared the immunoexpression of p53 in SA, UA, and OKC (16), and SA and OKC (17,18). Interestingly, other studies reported higher levels of p53 in OKC, followed by SA and UA (15), suggesting that the elevated p53 expression in OKC and SA reveals similarity in their locally aggressive biological behaviour (15,16).

Several studies have analysed the expression of p53 protein in UA (15,16), however, analysis by type/variety of UA has been described in only a few of these studies (19). The evaluation of the molecular expression by type/variety of UA should be carefully considered because of the differential clinical-pathological behaviour and treatment, particularly with lesions exhibiting mural growth.

Indeed, we found the highest levels of p53 in SA, MUA, and I/LUA. Other studies have reported elevated levels of p53 protein in UA compared to SA, especially for histological types of UA with mural proliferation (19). These data suggest that the centrifugal growth patterns from the luminal epithelium would be related to abnormal levels of p53 in UA.

A distinctive molecular expression pattern has been described for syndromic and non-syndromic OKC (7). The overexpression of p53 protein has been described with statistical significance in OKC-NBSCC compared to OKC-NS/S (20). Nevertheless, in our study no significant difference was observed between OKC-NBSCC and OKC-NS/S. Similar results are observed in other studies (21). Notably, immunolocalization parameters of p53 protein were observed at basal and suprabasal compartments of the epithelial lining of OKC. Immunodetection in the suprabasal compartment would be related to a longer G1 phase (21). Therefore, the biological behaviour of OKC may be related to the suprabasal proliferative compartment in the cystic epithelium. These dates suggested that a higher rate of epithelial lining proliferation is associated with p53 overexpression (22). Overall, the increase of inactivated p53 in odontogenic tumours and cysts induces impairment of the control of cell cycle, leading to an intrinsic growth potential that would be related to the potential locally aggressive behaviour (15,22).

In our study, immunoexpression of Bcl-2 protein was observed mainly in the basal compartment of the epithelial lining for OKC-NS/S and OKC-NBSCC and in ameloblast-like cells for CA and UA. Higher levels of the anti-apoptotic protein Bcl-2 suggest that inhibition of apoptosis could promote increased cell proliferation in these epithelial cell populations. Likewise, greater immunoexpression of Bcl-2 protein were detected in CA and UA compared to OKC-NS/S and OKC-NBSCC. In other studies, ameloblastoma showed stronger Bcl-2 protein expression than OKC (23), and higher expression of Bcl-2 protein in CA compared to UA (24), while, in a study for OKC-NS/S and OKC-NBSCC, Bcl-2 did not show significant difference (20).

Although investigations about the expression of the p53 and Bcl-2 detected by immunohistochemistry in tumours and cyst odontogenic are extensive (15-17,19-21,22), studies about Bax protein expression in odontogenic lesions are scarce (17,23-25). Similar to our study, González-González *et al*. (24) detected higher expression of Bax protein in MUA compared to LUA and IUA. We suggest that lower expression of the pro-apoptotic Bax protein in the luminal epithelial lining of UA could be related to tumour proliferative potential and cell growth towards the cystic cavity and the cystic wall.

Various studies described the expression of Bax protein in OKC (17,23,25), but they exclude the cases associated with NBSCC. In our study, the expression of Bax in OKC-NBSCC was lower than in OKC-NS/S. Eventually, the context of p53 overexpression and reduced expression of proapoptotic factors (Bax) would participate in the tumour development of OKC. However, some studies suggest that the exclusive analysis of Bax protein expression does not reflect the biological behaviour for OKC (25). Indeed, tumour cells can acquire resistance to apoptosis by subexpression and/or mutation of Bax or by the overexpression of Bcl-2, both proteins regulated by p53 gene (4).

P53 participates directly in the intrinsic apoptosis pathway by interacting with the multidomain members of the Bcl-2 family to induce mitochondrial outer membrane permeabilization (2,3). P53 stimulates Bax and inhibits Bcl-2 (anti-apoptotic protein from Bcl-2 family). Bax can dimerize with itself or Bcl-2, and when it is overproduced, Bax homodimers promote apoptosis. In contrast, when Bcl-2 is in excess, Bcl-2 homodimers predominate and cells are protected from death (26). Consequently, the ratio Bcl-2/Bax represents a cell death switch, which determines the viability of cells in response to an apoptotic stimulus; i.e. when Bcl-2/Bax ratio decreases, the cellular resistance to apoptotic stimuli also decreases, leading to increased cell death and reduced incidence of tumours (27). In our study we did not observe significant differences in the Bcl-2/Bax ratio between the lesions analyzed. Nevertheless, we identified that all morphological areas of UA presented a trend for Bcl-2/Bax ratio >1, which may indicate a similarity of the apoptosis dysregulation in UA. Moreover, Zhang *et al*. (28) describes that a Bcl-2⁄Bax ratio>1 prevents spontaneous apoptosis in oral squamous cell carcinoma, which leads to an accumulation of tumour cells. In this sense, the determination of the Bcl-2/Bax ratio could be indicated in the assessment of the dysregulation of apoptosis in odontogenic lesions.

Among the limitations should be considered that: Our evaluation was based on a descriptive study and does not allow a functional evaluation of these proteins, future investigations could complement our findings through other techniques, and the follow-up of patients and the consolidation of other clinical information is limited by the diagnostic characteristics of our research centre.

## Conclusions

The effect of mutant p53 activity over Bcl-2 and Bax expression could be relevant for the development and growth of odontogenic tumours and cysts. There is a tendency for an increased expression of p53 and Bcl-2 in solid tumours (CA) and focal areas of mural ameloblastomatous proliferation for UA compared to lesions with a cystic morphology (OKC and LUA). Further investigation is required to elucidate the interactions between both proteins and their role in the pathogenesis of odontogenic lesions.
